# Considering severity in the design of reproductive genetic carrier screening programs: screening for severe conditions

**DOI:** 10.1038/s41431-024-01738-0

**Published:** 2024-11-25

**Authors:** Lucinda Freeman, Alison D. Archibald, Lisa Dive, Martin B. Delatycki, Edwin P. Kirk, Nigel Laing, Ainsley J. Newson

**Affiliations:** 1 Graduate School of Health, University of Technology Sydney, Sydney, NSW Australia; 2 Victorian Clinical Genetics Service, Murdoch Children’s Research Institute, Melbourne, VIC Australia; 3 Sydney Health Ethics, Sydney School of Public Health, Faculty of Medicine and Health, The University of Sydney, Sydney, NSW Australia; 4 Murdoch Children’s Research Institute, Parkville, VIC Australia; 5 School of Women’s and Children’s Health, UNSW, Randwick, NSW Australia; 6 Centre for Clinical Genetics, Sydney Children’s Hospitals Network NSW, Sydney, NSW Australia; 7 NSW Health Pathology East Genomics, Randwick, NSW Australia; 8 Centre of Medical Research, The University of Western Australia, Perth, WA Australia; 9 Harry Perkins Institute for Medical Research, Perth, WA Australia

**Keywords:** Population genetics, Ethics

## Abstract

Reproductive genetic carrier screening (RGCS) provides information about people’s chance of having children with certain genetic conditions, to inform reproductive decision making. RGCS at population scale requires a robust and streamlined program that is purposively designed and formally implemented to ensure equity and consistency. There are many considerations in selecting conditions, genes and variants for inclusion in RGCS, with severity of the genetic condition a key criterion. However, the concept of severity is complex and often underspecified in available guidelines. Severity is often determined in relation to other contextual features and can be experienced differently by individuals who all have the same condition. While some genetic conditions are unambiguously considered severe, there are many factors that contribute to how severe a condition is perceived to be (and by whom), and perspectives will vary. In this paper, we analyse why severity is an important criterion when selecting conditions, genes or variants to be included in RGCS. We suggest that screening programs should be oriented more towards variants and genes associated with severe conditions. We discuss the importance of taking a practical approach to gene selection in a carrier screening program when presenting the offer at population scale.

## Introduction

Recent years have seen significant advances in the use of genomic technologies for reproductive genetic carrier screening (RGCS, also known as expanded or universal carrier screening when large numbers of genes are tested) [1, 2]. Broadly, RGCS involves screening a reproductive couple (the two individuals of female and male sex who are or will be the genetic/biological parents) either simultaneously or sequentially to ascertain whether there is an increased chance that their offspring will have, or develop, certain autosomal recessive and/or X linked conditions. RGCS is available in some countries, either on a user-pays basis through commercial providers, or via government funded or subsidised offers. While it does raise ethical issues, research on RGCS has demonstrated strong patient and public support [3–6].

As genomic technologies advance and knowledge increases, RGCS is likely to become more widely accessible. With such increasing access alongside greater demand, it is therefore likely that it will be offered at population scale as a formal population screening program in many countries. That is, we can expect RGCS to become a more routine part of reproductive planning, regardless of a person’s genetic background, ethnicity or family history. While RGCS can be provided in numerous ways, including through individually initiated commercially provided testing, in this paper we focus on formal offers through publicly funded avenues such as population screening programs.

We aim to address the complex decision-making required when offering RGCS at a population level. Given this focus, instead of providing an indicative list of conditions or suggesting a categorisation to enable a tier-based approach, our focus is rather on the way decisions are made when choosing what conditions to screen for and what variants to report. Whilst genomic testing technologies provide the technical capacity to screen a large number of genes at once, it is important to consider what type of genetic carrier information should be provided in the context of population-wide screening, including to those who have no known family history or no lived experience of a genetic condition. Thus, developing a scientifically robust, ethically defensible, feasible and consistent approach to RGCS is paramount.

Population screening guidelines have traditionally emphasised that strong evidence is needed to support the inclusion of any condition in a formal screening program [7]. This includes that the condition should be an important health problem, that its identification should be acceptable to the target population and that the condition itself should have a significant impact on the affected individual. However, as noted, genomic testing technologies enable screening of many genes in a single test. It has been argued that the characteristics of genetic testing require an augmented approach compared to the traditional criteria by which screening programs are justified [8–10]. As a result of advances in testing technology it will be increasingly (technically) feasible and potentially justifiable to screen for very rare genetic conditions at population scale. Wider offers of RGCS will also need to consider the reliability of variant interpretation for diverse groups within the population, particularly those under-represented in genomic datasets.

Given that end-user acceptability for RGCS is already generally satisfied, a key decision point for RGCS implementation therefore becomes the degree of impact or severity of a condition required to warrant inclusion in an RGCS gene panel. This consideration becomes even more important in the context of a government or publicly funded RGCS program because policy-makers need to ensure the included conditions are acceptable to wider society without causing harm.

Severity can be broadly defined as a measure of the extent to which a genetic condition impacts on the life of an individual with that condition [11]. In addition to the broad public support for RGCS already noted, research also shows strong support for screening for severe conditions in the context of such programs. However, this support decreases with decreasing perceived severity of the conditions included in the program [12–14]. Further, prenatal diagnosis for mild conditions or conditions with variable and/or uncertain clinical impacts appears to be associated with greater decisional conflict and other forms of distress [15, 16]. There is also discomfort with the notion of screening for genetic information associated with mild clinical impacts or genetic changes that are considered to be part of usual person-to-person variation, such as eye color [17]. Using such information to inform reproductive decision-making can be perceived as concerning in light of moves towards the elimination of disability and difference, and because such decisions may have the potential to influence future generations [13, 18]. Thus, the greatest societal acceptance of, and ethical justification for RGCS can arguably be achieved when the focus is on screening for severe conditions, namely those which have a significant impact on the individual with the condition and their family.

In this paper, we build on our previous theoretical work to conceptualise severity [19] by proposing how considerations of severity can be operationalised in the practical context of a population-level offer of RGCS. A population-level offer of RGCS is, for present purposes, considered comparable with a government funded RGCS program. We begin by describing the complexity of choosing which genes and conditions to include in an offer of RGCS, and the role that severity plays. Here, we highlight important practical considerations for determining the degree of severity. In the second part of the paper, we address issues related to variant interpretation and consider the implications of severity for RGCS program design.

## Severity as an inclusion criterion for RGCS

International guidelines and recommendations consistently emphasise the importance of informed choice in RGCS and the need for implementation to achieve an equitable, accessible, and responsible offer to the population [20–22]. These recommendations are helpful in providing guidance to those offering RGCS. However, despite these endorsements to offer RGCS, there remains no agreement on which genes and variants should be included in the offer, nor how decisions should be made on such inclusion. Whilst guidelines typically include clauses indicating that conditions screened for should be severe and have a significant impact on an individual’s quality of life, there is a lack of clarity around what constitutes a ‘severe’ condition and how to measure the impact on a person’s quality of life [20, 23]. RGCS is often described as screening for severe, childhood onset conditions, or for conditions for which couples may reasonably expect to consider changing their reproductive planning [24, 25]. However, the 2021 American College of Medical Geneticists (ACMG) practice resource [22] suggests including common conditions (with a carrier frequency of at least 1 in 200) that are at least moderately severe, as assessed by a taxonomy based on clinical impact [26].

Several studies have presented the complexities associated with determining boundaries around which conditions to include in RGCS, particularly when it comes to including conditions characterised by varying presentations, onset in later years and milder phenotypes [11, 21, 27]. The decision as to whether a condition is severe enough to be included in RGCS has inherent epistemic challenges, including how to account for subjective experience and knowledge. The perception of severity associated with a condition can be influenced by numerous factors and can differ depending on whose perspective is considered: a person living with the condition; family members of a person living with the condition; clinicians who treat individuals with the condition, and so on. Societal norms about disability and difference also exert an influence on how the lives of people with certain conditions are perceived [28]. Severity is not a simple, scalar property, it consists of various dimensions that will have varying emphases, depending on context.

One additional point to make regarding the offering of RGCS in different countries is the need to consider regional and/or cultural differences regarding severity. Such differences are likely to exist within as well as between populations. It is vital to offer RGCS in a way that attends to cultural safety for all people. The experience of severity of a condition is likely to also have a cultural component which would add another layer of complexity to the experiential narratives.

Given the intensely personal nature of reproductive decisions made following receipt of RGCS results, it has been argued that the composition of gene panels should not be determined solely by clinicians but should also incorporate views of a wide group of stakeholders, including those who would use this testing (prospective parents) and those who have a lived experience of the conditions being considered [29]. It is crucial to incorporate diverse perspectives on severity, since the inclusion of a condition in an offer of population screening has the capacity to influence how people with such conditions are perceived [30]. The taxonomy for severity cited by the ACMG in their practice resource uses a set of criteria to assess and categorise severity of genetic conditions into profound, severe, moderate and mild based on clinical perspectives of the impact on an individual’s health and function [26]. However, these criteria are considered and applied using the expertise of genetic healthcare professionals.

To be defensible, we contend that any categorisation or taxonomy of severity needs to satisfy several elements. These include that the categorisation should incorporate and respond to the ethical complexity of defining severity, must include testimony from those with lived experience, and should avoid relying predominantly or only on clinical factors to decide whether a condition is ‘severe’ enough to be included in RGCS. While the taxonomy noted above has the advantage of allowing a consistent approach, it has important limitations as we have explored previously [19].

A further challenge in designing a screening panel is whether to include conditions that may be considered serious and with childhood onset, but which have variable expressivity and incomplete penetrance. There are numerous examples of conditions which have a spectrum of severity ranging from lethality in infancy through to mild disease with onset in adulthood, or even non-penetrance. An important example is the spectrum of phenotypes associated with *CFTR*, ranging from classical cystic fibrosis through to congenital bilateral absence of the vas deferens and/ or mild sinopulmonary disease in adults. For such conditions, we have previously highlighted the need for inclusion to reflect the likely value of the genetic information to inform reproductive decision making [31]. The question of what information is beneficial in reproductive decision making is complex and remains open [31, 32].

A recent study in an Australian population found that parents of children with genetic conditions were open to seeking RGCS; however, as the conditions became clinically milder, support for their inclusion decreased [12]. That study also reported that participants who indicated that they would use reproductive options to avoid having children with the condition similarly decreased in number as the conditions became clinically less severe [12]. Studies have also found that couples with an affected pregnancy consider the reported severity of a condition in their reproductive decision making [25, 33, 34]. In the context of population RGCS, many couples will not have had any personal experience of the conditions being screened. A challenge for many couples in making a decision based on severity is that determining severity requires a judgment about the possible impact of that condition on their future child in the context of their own family structure and dynamic [35].

There are other challenges in assessing severity beyond the experiential and clinical management. Whilst many conditions are caused by variants in a specific gene, some genes are associated with multiple clinically distinct conditions (i.e., pleiotropy) and some conditions can be caused by variants in multiple different genes. Thus, when developing a RGCS panel, it is important to assess the severity of each gene-condition pair separately. However, the frequent variability in severity can make such classifications challenging. Consideration must be given to the likelihood of the most severe phenotype and the most likely phenotypic outcome. Where there are treatments or preventive interventions available for the condition, they can vary in terms of effectiveness, accessibility, and how burdensome the treatment is to the child and family. With access to RGCS increasing, it is essential to approach gene/ condition selection with robust and transparent approaches.

Given the potential societal implications of RGCS, any RGCS program needs to be established with broad community consultation, including people who may be offered screening, people who are impacted by the conditions, healthcare providers, ethicists, policy makers and the general community. The Australian Reproductive Genetic Carrier Screening Project (‘Mackenzie’s Mission’) took an approach akin to this, establishing a multi-sectoral gene selection committee to establish the initial gene list [36]. The challenge of navigating the likely differing views illuminates the need for appropriate community engagement beyond simply reporting stakeholder views. A population-wide RGCS offer will never be able to satisfy diverse participants’ individual preferences, but should strive for transparency and ethical justification in its inclusion criteria. Incorporating multi-sectoral consultation into RGCS program design will help to ensure the offer meets the needs of the population and that potential harms are assessed and minimised. With respect to severity, this broad input will be important in determining the threshold for a gene to be included in the screening.

## Incorporating severity considerations into RGCS program design

A cautious and deliberative approach is crucial for incorporating considerations of severity into designing and implementing RGCS. Severity considerations can be addressed at each stage of screening program delivery. Proactive engagement with consumer support organisations representing conditions included in RGCS is paramount to ensure responsible design and implementation of screening.

### Pre-test

Recognising that most people will not have experience with either RGCS or the conditions screened, RGCS programs should start by engaging possible screening participants with the rationale for screening, including the part that severity played in program design as well as what outcomes it might lead to. Programs should also include high quality balanced information about included conditions. Conveying the lived experience of screening and what it is like to have one of the conditions screened is vital so people can personalise and relate to the experience and the information it will generate. As many hundreds of conditions can be included in RGCS, it is neither appropriate nor possible to describe each condition. However, conditions can be grouped into categories with creative approaches to information delivery such as infographics, videos and/or audio recordings describing the types of conditions screened. Decision aids can be beneficial in helping people to consider how RGCS for the included conditions aligns with their beliefs and values and encourages both members of the reproductive couple to engage in this deliberation [37]. The option to speak to a genetic counselor is also useful for those who have specific questions, require more detailed information or are seeking decision-making support.

### Testing

As outlined above, severity is a key factor in selecting genes to screen. It also must be considered at the analysis/ reporting phase of testing. As a high degree of confidence is needed to inform reproductive decision-making, for RGCS the focus should be on reporting clinically significant variants: class 4 (likely pathogenic) and class 5 (pathogenic). Reporting variants of uncertain significance—while considered permissible by some professional bodies – has low reproductive utility, creates complex decision-making and may lead to unnecessary reproductive interventions [22, 38]. As screening approaches are targeting populations with low a priori risk of the conditions, there is usually no clinical information (such as phenotypic information, or family history) available to help guide decisions about variant classification. Thus, variant classification is based on a subset of the categories of evidence usually used for this purpose. For variants that are not predicted to cause loss of function, classification as at least likely pathogenic is only possible if there are reports of affected individuals with the variant [38].

For many genes included in RGCS, different pathogenic or likely pathogenic variants can have different impacts on gene and protein function, which in turn can cause variability in clinical presentation. A key challenge with reporting RGCS results is that a gene may be included in an RGCS panel due to its association with a severe phenotype, but variant combinations may occur that cause a milder form of the condition. Assessment of predicted severity draws on some of the same data used to classify a variant in the first place but requires additional analysis and consideration.

Nevertheless, reporting *all* class 4 and 5 variants in RGCS can be problematic as variant combinations associated with mild clinical presentations may have low reproductive utility. Thus, a second step in the analysis/reporting process can be helpful to enable consideration of the severity of the particular variant combination. Reporting of variant combinations associated with severe clinical presentations ensures results have reproductive utility. In Mackenzie’s Mission a variant review committee considered the suitability of variant combinations for reporting considering both variant pathogenicity and severity [38].

Given the subjective nature of severity, a multidisciplinary and collaborative approach to report decision-making enables a balanced approach to result reporting. Using a two-step approach to assessing variants minimises reporting of results conveying risk for mild conditions, which in turn reduces reproductive decision-making complexity and allows clinical resources to focus on reproductive couples who have an increased chance for a severe condition in their offspring. However, assessing the likely severity of the phenotype associated with a variant combination can be very challenging, particularly for very rare conditions for which there is limited published evidence available. As noted, screening for ultra rare conditions is possible and often appropriate. However, counseling of couples about such conditions may be made more challenging due to limited evidence regarding the expected range of severity associated with a given variant or variant combination, and limited ability to draw on the experiences of those with these conditions and their families. Moreover, some variants are associated with phenotypes of widely varying severity, and decisions may need to be made to report based on the most likely or most severe potential outcome, depending on context and frequencies. For example, the common ‘S allele’ in the *SERPINA1* gene (associated with alpha-1 antitrypsin deficiency) is classified as pathogenic as it can cause clinical disease if inherited with the common and severe ‘Z allele’. However, the PI*SZ genotype is not associated with severe childhood liver disease, and penetrance for adult onset lung disease is incomplete and mainly associated with environmental triggers such as smoking. Thus, whilst it is pathogenic, the S allele has low utility in the reproductive context as it is not associated with a severe clinical presentation. As a result, it is unlikely to prompt the choice to access reproductive interventions to avoid the birth of a child with that genotype [39].

RGCS is rapidly developing and a commonly used approach to variant reporting has been to report any potentially clinically significant variants. Approaching reporting in this way has generated large volumes of results, many with limited reproductive utility. Such results cause confusion among those having screening and some health care providers, introduce additional complexity into decision-making and, in some cases, lead to unnecessary reproductive intervention (unpublished data). A clearly established and consistent variant reporting approach tailored to the RGCS context is needed. Professional guidelines outlining standards of care in RGCS would assist with consistency in service delivery, minimize negative psychosocial impacts, and optimize use of clinical resources.

### Post-result

Reproductive couples receiving a result indicating an increased chance that they will have a child with a severe childhood onset genetic condition are unlikely to have knowledge or experience of the condition. This result is very important: it will inform decisions about whether to undergo further intervention to avoid the birth of a child with this condition. As such, couples must have access to support and information about the condition and how it aligns with their values and hopes for parenthood. Providing an accurate and balanced representation of the condition will help to ensure they are able to make informed decisions. Online information tends to focus on clinical aspects of the condition, which can make it difficult for people to develop a clear sense of what it is actually like to raise a child with it, not to mention what life might be like for the child themselves. Receipt of an increased chance result very understandably will lead a person to seek further information, and the first method for so doing is usually an online search. The results that are returned in that search need to be such that they address current limitations. RGCS providers should work together to ensure that high-quality, holistic resources are developed and widely shared.

Genetic counseling can also be instrumental in helping people understand and adapt to their result. It can help couples with increased chance results understand the relevant condition, by offering clear and relatable descriptions of the condition, linking people in with a relevant patient support organisation for the condition and/or providing referral to healthcare providers with expertise in the condition. Other tools such as decision-aids to support reproductive decision-making and interactive tools providing insight into day-to-day life with the genetic condition could be developed to further support this process [37].

## Conclusion

Most couples who participate in RGCS do so because they are seeking information relevant for their reproductive decision making. In the context of a formal RGCS program, couples rely on decision makers to have chosen conditions (and genes, variants or variant combinations that result in those conditions) that can best achieve the outcome of informed reproductive decisions. To meet this need, we have articulated the rationale for programs to focus on severe conditions. Designing programs to include this criterion will help minimise ambiguity and uncertainty for those both providing and using RGCS. It is also important that any program has comprehensive decision-making supports in place for those who need them following a RGCS result.

## Data Availability

Not applicable as no data was analysed in this manuscript.
